# Integrating Wind Speed Into Climate‐Based West Nile Virus Models: A Comparative Analysis in Two Distinct Regions

**DOI:** 10.1029/2024GH001320

**Published:** 2025-07-05

**Authors:** Eric R. Bump, Anita Bharadwaja, Sean Simonson, Emma Ortega, Michael C. Wimberly

**Affiliations:** ^1^ University of Oklahoma Norman OK USA; ^2^ South Dakota Department of Health Pierre SD USA; ^3^ Louisiana Department of Health Baton Rouge LA USA

**Keywords:** vector‐borne disease, West Nile virus, climate, wind speed

## Abstract

Since its introduction to North America in 1999, West Nile virus (WNV) has become the most widespread mosquito‐borne disease in the United States. Climatic conditions significantly influence transmission dynamics. While temperature, precipitation, and humidity are known to affect mosquito populations and virus replication, wind speed is often neglected in transmission models despite its potential to alter mosquito behavior and facilitate mosquito dispersal. This study incorporates wind speed into climate‐based WNV models to compare its effects in Louisiana and South Dakota, two U.S. states with contrasting climates, land cover, and vector and host species. From 2004 to 2022, we analyzed weekly WNV human case data in relation to daily meteorological data. The relationships were modeled using logistic regression with distributed lag effects. Incorporating wind speed consistently enhanced the fit of climate‐based models across both states, as evidenced by the Akaike Information Criterion. Higher‐than‐normal wind speeds were associated with decreased WNV cases over specific lag periods, suggesting that increased wind speed may inhibit mosquito activity and reduce virus transmission. Differences in how temperature and moisture‐related variables influenced the two regions highlight the importance of considering regional climatic contexts. These findings demonstrate that incorporating wind speed can enhance meteorological models of mosquito‐borne diseases and reinforce the importance of considering a broader range of climatic factors beyond temperature and precipitation. Understanding these regional variations is essential for predicting local climatic influences on disease transmission, which can support the implementation of more targeted and effective public health strategies.

## Key Points

- Integrating wind speed into climate models consistently improved model performance for WNV cases in Louisiana and South Dakota
- Higher than normal wind speeds decreased reported WNV cases in both Louisiana and South Dakota
- Climate variables exhibited different influences on WNV incidence between the two states, underscoring the importance of regional context

## 1 Introduction

First isolated in 1937 in the West Nile district of Northern Uganda, West Nile virus (WNV) was historically confined to Africa, the Middle East, and parts of Europe (Chancey et al., 2015; Heidecke et al., 2023). Its emergence in New York City in 1999 marked a pivotal change in its geographical distribution. Following this introduction, WNV spread rapidly across the United States, eventually becoming the nation's leading mosquito‐borne disease (Lanciotti et al., 1999; Roehrig, 2013). In North America, WNV is primarily transmitted by mosquitoes of the genus *Culex*, with *Culex pipiens*, *Culex quinquefasciatus*, and *Culex tarsalis* serving as the main vector species across different regions (Kilpatrick & Pape, 2013). Mosquitoes acquire WNV by feeding on infected birds, a critical step for viral amplification and maintenance in nature. The diversity, abundance, and migratory behaviors of avian hosts significantly influence local virus transmission dynamics (McLean et al., 2001). Once infected, mosquitoes can transmit WNV to humans and other animals during subsequent blood meals (Hayes et al., 2005; Reisen, 2013). However, humans and most mammals do not develop sufficient viremia to continue the transmission cycle, classifying them as incidental or “dead‐end” hosts (Heidecke et al., 2023). Human WNV infections present a broad clinical spectrum, from asymptomatic to severe neuroinvasive conditions such as encephalitis and meningitis. Severe cases occur in fewer than 1% of infections but can be life‐threatening (Petersen et al., 2013). Between 2005 and 2019, annual reported WNV cases in the U.S. varied widely, from approximately 700 to 6,000, reflecting the sporadic and unpredictable nature of outbreaks influenced by ecological and climatic factors like vector dynamics, bird migration, and weather conditions (Gorris et al., 2023; Reimann et al., 2008; Smith et al., 2020). A significant number of asymptomatic infections complicates accurate disease surveillance and masks the true extent of virus spread, highlighting the need for effective vector management and preventive strategies (Gubler et al., 2001; Hongoh et al., 2016; Wang et al., 2024).

Since its introduction to the U.S., WNV has demonstrated substantial geographic and temporal variability, largely driven by climatic factors affecting mosquito and bird populations (Epstein, 2001; McLean et al., 2001; Tolsá et al., 2018). Mosquito life cycles, highly sensitive to environmental conditions, underscore the importance of understanding interactions between ecological and climatic factors in WNV transmission (David & Abraham, 2016; Kain & Bolker, 2019; Wang et al., 2024). Key climatic drivers include temperature, precipitation, and humidity, each uniquely impacting mosquito development and behavior (Brown et al., 2023; Morin & Comrie, 2013; Paz, 2015; Reisen et al., 2008). Warm temperatures, stagnant water, and high humidity create ideal conditions for Culex mosquitoes. Species such as *Cx*. *pipiens* and *Cx*. *quinquefasciatus* typically breed in stagnant water with high organic content, including eutrophic or polluted environments, while *Cx*. *tarsalis* prefers cleaner habitats with moderate nutrient levels, such as irrigation ditches and wetlands (Chase & Knight, 2003; Kilpatrick et al., 2008; Shaman et al., 2005; Why et al., 2016, 2021).

Mosquito vectors of WNV, such as those from the Culicinae subfamily, are ectotherms, meaning their body temperatures fluctuate in response to external environmental conditions. As a result, key physiological processes such as blood meal digestion, life‐stage development, survival, and viral replication are highly sensitive to ambient temperatures, influencing mosquito population dynamics and determining WNV geographic range (Kristan et al., 2018; Mordecai et al., 2017; Shocket et al., 2020). Higher temperatures accelerate mosquito development and viral replication, reducing the incubation period before mosquitoes become infectious and shortening intervals between blood meals, thus enhancing transmission potential (Dohm et al., 2002; Mordecai et al., 2017; Shapiro et al., 2017; Turell et al., 2001). However, excessive heat imposes biological constraints, reducing the longevity and reproductive success of mosquitoes. Rapid development at warmer temperatures often results in smaller mosquitoes with decreased survival and lower reproductive capacity, limiting the sustained population necessary for effective WNV transmission (Barr et al., 2023; Mordecai et al., 2019). WNV transmission peaks at temperatures between 23°C and 26°C, with optimal transmission near 24°C. This unimodal pattern illustrates how transmission efficiency declines at both cooler and hotter extremes, highlighting the critical role of temperature in shaping disease dynamics (Shocket et al., 2020). Understanding these temperature‐dependent processes is crucial for developing effective vector control strategies and anticipating changes in disease transmission under climate change scenarios.

Hydrological conditions can modulate WNV transmission by shaping breeding environments for Culex mosquitoes. Although rainfall is crucial for creating the stagnant water bodies necessary for mosquito larvae development, both excessive and insufficient precipitation can negatively impact mosquito survival. Heavy rains can flush out breeding habitats, thereby reducing mosquito populations (Caldwell et al., 2021; Koenraadt & Harrington, 2008; Tjaden et al., 2018). Conversely, prolonged dry conditions often prompt human behaviors such as water storage and irrigation, which inadvertently create artificial breeding sites, sustaining mosquito populations in the absence of natural aquatic habitats (Landesman et al., 2007; Lowe et al., 2021; Trewin et al., 2013). Furthermore, excessive rainfall can inundate mosquito breeding sites, decreasing larval survival rates and disrupting their life cycles (Benedum et al., 2018). Seasonality also significantly affects these processes, as the timing and intensity of rainfall determine mosquito population dynamics and influence WNV transmission patterns (Morin & Comrie, 2013). Adequate rainfall during winter and spring establishes breeding habitats, while subsequent warm, dry conditions in summer accelerate mosquito development and concentrate mosquito and bird populations, thereby enhancing the risk of WNV transmission (Shaman et al., 2005, 2011).

Although humidity can strongly affect mosquito ecology, it has often received less attention compared to temperature and precipitation, possibly due to the absence of robust conceptual models to quantify its effects (Brown et al., 2023). High humidity promotes precipitation and reduces evaporation, facilitating the accumulation and maintenance of standing water, thereby increasing the availability of mosquito breeding habitats. Maintaining moisture in these sites creates optimal conditions for egg‐laying and larval survival (Thomas et al., 2018; Yamana & Eltahir, 2013). Additionally, humidity directly impacts mosquito thermoregulation, influencing survival, reproduction, and vector competence. Elevated humidity levels reduce the risk of desiccation, allowing mosquitoes to conserve water and extend their lifespan, particularly in environments where dehydration would otherwise limit survival (Rozen‐Rechels et al., 2019). By decreasing energy demands associated with thermoregulation and water balance, higher humidity can prolong mosquito lifespans, increase population sizes, and consequently elevate the risk of WNV transmission (Benoit & Denlinger, 2010; Brown et al., 2023; Drake et al., 2010).

Despite its potential to shift mosquito behavior and disease transmission by affecting their flight capabilities, wind speed remains an understudied factor in climatic research on mosquito‐borne diseases. This environmental variable can shape mosquito dispersal and distribution, potentially introducing pathogens into new areas and populations (Atieli et al., 2023; Endo & Eltahir, 2018a, 2018b; Lehmann et al., 2023). High wind speeds can inhibit mosquito flight, suppress blood‐feeding behaviors, and reduce disease transmission rates. During calmer conditions, mosquitoes may increase feeding activity to compensate for previous restrictions (Gillies, 1961; Service, 1980). In addition, prevailing wind patterns can guide mosquito populations toward densely populated or high‐risk areas, potentially intensifying the spread of WNV (Paz, 2015).

Several previous studies have documented the influences of wind on WNV mosquito vectors and human disease. Ferraccioli et al. (2023) reported that moderate wind speeds enhance mosquito dispersal, increasing transmission potential, while high winds diminish mosquito activity and reduce transmission rates. Similarly, Stilianakis and Sudre (2016) found that higher wind speeds negatively affected human WNV cases and the presence of WNV‐infected mosquitoes. Rehbein et al. (2024) investigated the impact of wind speed, temperature, and precipitation on *Culex* species abundance and WNV infection rates in rural Illinois. Their findings suggest that low to moderate winds enhance mosquito dispersal and virus transmission, while high winds suppress activity and reduce infection rates. Lapointe (2008) demonstrated that wind facilitates long‐distance dispersal of *Cx*. *quinquefasciatus* in Hawaiian rainforests, increasing WNV risk in previously unaffected areas. Cummins et al. (2012) examined mosquito population dynamics, demonstrating that wind significantly influences mosquito dispersal and host‐seeking behavior, with different wind‐driven flight strategies affecting their success in locating hosts. These studies highlight the importance of incorporating wind into WNV models, alongside other climatic variables, to improve predictive accuracy (Heidecke et al., 2023; Ward et al., 2023). Paz (2015) highlighted that climate change could alter wind patterns, potentially expanding WNV's geographic range by shifting the dispersal patterns of mosquito vectors and influencing avian migration routes.

This research aims to integrate wind speed into climate‐based models of WNV incidence to clarify its influence on disease dynamics alongside temperature, precipitation, and humidity. Despite its potential impact, wind speed remains underexplored in studies of mosquito behavior and disease transmission (Ellwanger & Chies, 2018). This study addresses this gap by investigating the effects of wind speed on WNV cases and comparing these impacts between two distinct states, Louisiana and South Dakota. Specifically, the research is guided by two central questions: (a) How does incorporating wind speed affect climate‐based models of WNV? (b) Are the effects of climatic anomalies on WNV incidence consistent across different regions, or do they vary due to region‐specific environmental contexts?

## 2 Methods

### 2.1 Study Area

**Figure 1:**
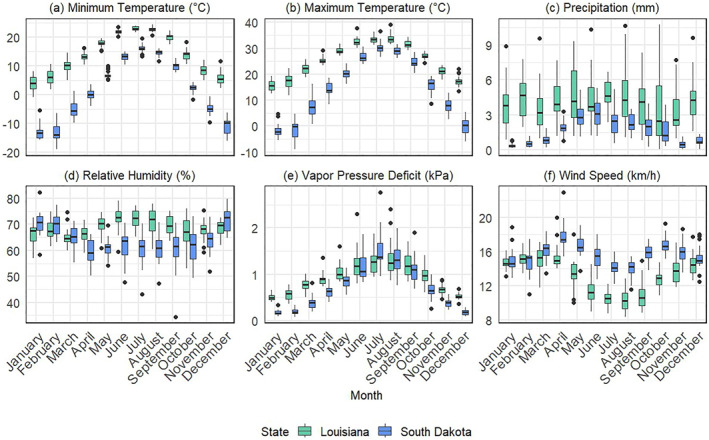
Interannual variation from 2000 to 2023 in monthly climate variables for Louisiana and South Dakota: (a) Minimum Temperature (°C), (b) Maximum Temperature (°C), (c) Precipitation (mm), (d) Relative Humidity (%), (e) Vapor Pressure Deficit (kPa), and (f) Wind Speed (km/h). Each boxplot shows the distribution of monthly county‐level means derived from daily GridMET data, with Louisiana in green and South Dakota in blue. The black dots represent outliers beyond the whiskers of each boxplot, highlighting the range and variability of climatic conditions across the two states.

Louisiana and South Dakota each exhibit distinct climates, ecosystems, and vector–host dynamics, making them valuable comparative settings for examining climate‐driven WNV epidemiology (Figure 1). They also differ substantially in human population size, density, and land cover factors that may influence WNV surveillance, reporting, and transmission (Heidecke et al., 2023; Smith et al., 2020).

Louisiana is situated in the humid subtropical zone of the southeastern United States (Vega et al., 2012). The state's climate varies from cooler, less humid conditions in the north to warmer, more humid environments along the Gulf Coast. Average minimum temperatures in January are approximately 4.0°C, while July maximum temperatures average around 33.5°C. Annual precipitation is about 1,500 mm, and mean relative humidity is 68.7%, with higher rainfall and moisture concentrated in southern parishes (Baril et al., 2023; Gorris et al., 2023; Moise et al., 2018, 2021). Land cover is notably diverse, with the northern and central regions characterized by mixed forests (deciduous and evergreen), pasture/hay, and cultivated crops (U.S. Geological Survey, 2023). Deltaic and coastal zones feature extensive wetlands, particularly around the Atchafalaya and Mississippi basins, that, combined with mild winter temperatures, facilitate year‐round mosquito activity. These conditions favor *Cx*. *quinquefasciatus* (the Southern house mosquito). Demographically, Louisiana has a population of approximately 4.6 million (U.S. Census Bureau, 2020), with 17.4% aged 65 or older. Population density is 41.6 people per square kilometer, with 74% residing in urban areas, which are factors that can affect both mosquito–human contact rates and surveillance coverage (Albrecht & Kaufeld, 2023; Smith et al., 2020).

South Dakota, located in the northern Great Plains, has a continental climate characterized by pronounced seasonal and interannual variability (Kibria et al., 2016). Winters are notably cold, with an average January minimum of −12.9°C, and summers reach a July maximum of 30.6°C. Annual precipitation averages around 590 mm, and mean relative humidity is about 63.8% in the eastern portion of the state. Grasslands and herbaceous vegetation dominate central and western regions, while cultivated crops prevail in the east (U.S. Geological Survey, 2023). These conditions support *Cx*. *tarsalis*, a vector species well‐adapted to fluctuating temperatures (Chuang & Wimberly, 2012; Reisen et al., 2006). South Dakota's total population of roughly 924,669 (U.S. Census Bureau, 2020) with 18.4% aged 65 or older, has a density of about 4.42 people per square kilometer, and is 56.5% urban. Compared with Louisiana, South Dakota's predominantly rural setting and lower urbanization may result in different disease reporting patterns and exposure pathways (Albrecht & Kaufeld, 2023).

Avian reservoir hosts play a critical role in the WNV transmission cycle, influencing both local virus amplification and geographic spread. Table 1 presents the most frequently observed bird species in Louisiana and South Dakota, emphasizing differences in potential reservoir communities (Eason et al., 2019). In Louisiana, American Crows (*Corvus brachyrhynchos*) and Blue Jays (*Cyanocitta cristata*) are particularly vulnerable to WNV infection and can develop sufficient viremias to infect mosquitoes (Mackay et al., 2014; Nguyen et al., 2019). Other passerines, including Northern Cardinals (*Cardinalis cardinalis*) and Common Grackles (*Quiscalus quiscula*), also become frequently exposed to WNV, though their peak viremias are more moderate. Field data suggest that *Cx*. *quinquefasciatus*, the dominant regional vector, primarily feeds on birds but will opportunistically feed on mammals bridging the virus to humans (Mackay et al., 2014). Louisiana's relatively mild winters allow substantial resident bird populations to remain in place, although direct bird‐to‐bird transmission of WNV outside of a mosquito vector appears limited (Hinton et al., 2015; Nguyen et al., 2019). Nevertheless, these overwintering hosts can potentially facilitate viral persistence until temperatures increase, at which point the force of infection can intensify with the renewed abundance of actively feeding *Culex* mosquitoes (Hinton et al., 2015; Mackay et al., 2014). Moreover, strong correlations in annual WNV case numbers between Louisiana and northern Great Plains states suggest that Louisiana acts as a “source” state from which infected migratory birds reintroduce WNV each spring (Schwartz et al., 2025).

**Table 1:** Top Ten Most Commonly Observed Bird Species in Louisiana and South Dakota (2004–2022), Based on Total Counts From the North American Breeding Bird Survey (Eason et al., 2019)

Louisiana			South Dakota	
Rank	Common name	Total count	Common name	Total count
1	Red‐winged Blackbird	57,676	Western Meadowlark	94,486
2	Northern Cardinal	54,965	Red‐winged Blackbird	58,838
3	White Ibis	33,776	Brown‐headed Cowbird	34,691
4	Cattle Egret	30,440	Mourning Dove	29,937
5	American Crow	29,971	Common Grackle	28,240
6	Northern Mockingbird	27,829	Lark Bunting	23,812
7	Carolina Wren	24,667	Cliff Swallow	23,446
8	Mourning Dove	23,797	Ring‐necked Pheasant	22,081
9	Blue Jay	19,802	American Robin	15,869
10	Common Grackle	19,759	Barn Swallow	11,564

*Note*. Species composition highlights distinct avian communities in each state.

In contrast, in South Dakota, *Cx*. *tarsalis* is the primary vector of WNV, with a strong feeding preference for certain passerines and periodic shifts to mammalian hosts later in the summer (Anderson et al., 2025; Kent et al., 2009). American Robins (*Turdus migratorius*) are often an early‐season focal host for *Cx*. *tarsalis*, providing enough viremia to initiate local amplification before tangential transmission to humans occurs (Anderson et al., 2025). Mourning Doves (*Zenaida macroura*) and Common Grackles (*Quiscalus quiscula*) are also bloodmeal sources in these areas, although their contributions to WNV cycling seem secondary (Kent et al., 2009). Due to significant changes in temperature and a greater proportion of migratory birds, WNV transmission in this region abates in cold months, with local virus activity resurging in spring after the return of migrating and possibly infected avian hosts (Anderson et al., 2025). The result is a pronounced seasonal pattern of transmission that contrasts with the potential for year‐round maintenance through resident birds in Louisiana (Mackay et al., 2014; Nguyen et al., 2019). Furthermore, states in the northern Great Plains, such as South Dakota, consistently emerge as WNV “hotspots” due to the interaction between large populations of breeding birds and highly competent vectors like *Cx*. *tarsalis*, creating conditions that intensify virus amplification (Schwartz et al., 2025).

**Figure 2:**
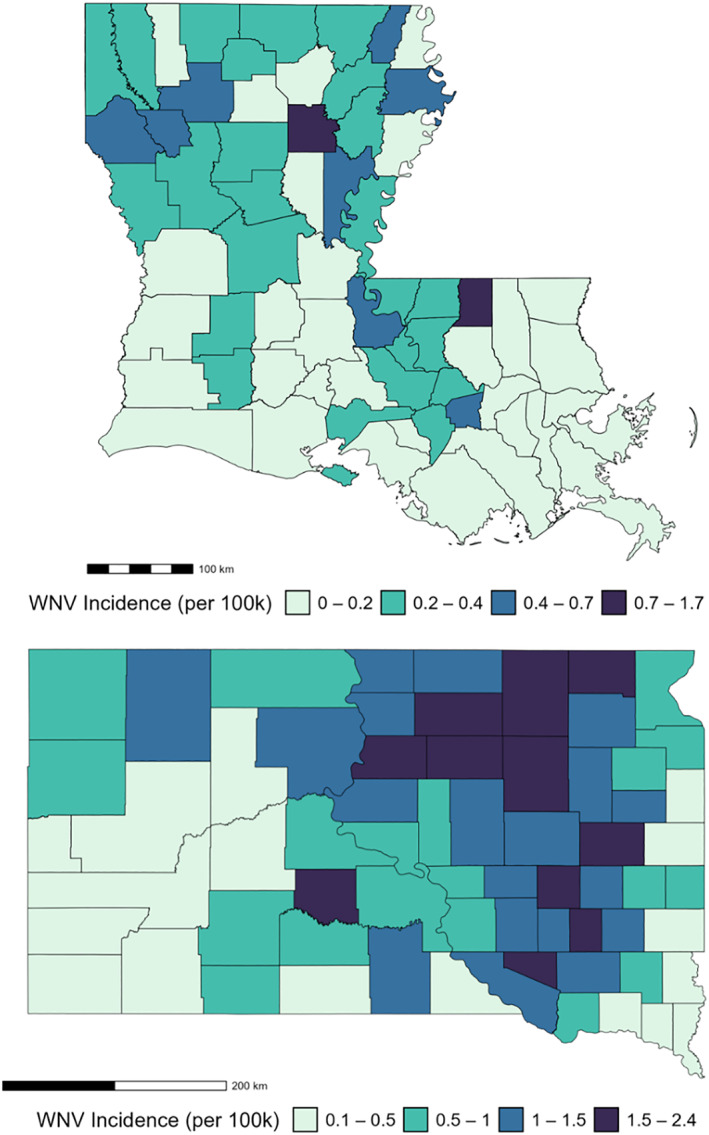
Occurrence of WNV in Louisiana (top) and South Dakota (bottom) from 2004 to 2022. Maps show the spatial distribution of WNV mean annual incidence per 100,000 population by county. Incidence values are based on the sum of WNV fever cases, neuroinvasive disease cases, and asymptomatic blood donations.

The interactions between vectors and hosts influence the transmission and dynamics of WNV in the distinctive climatic conditions of Louisiana and South Dakota, shaping the spatial patterns of its incidence (Figure 2). In Louisiana, WNV incidence is higher in northern and eastern regions. Similarly, higher incidence in South Dakota is concentrated in the northeast, particularly in the northern James River Valley. The reasons for these hotspots are multifaceted and may include a combination of factors such as environmental conditions, the presence of suitable mosquito habitats, the abundance of avian hosts, and human population distribution (Heidecke et al., 2023; Hess et al., 2018).

**Figure 3:**
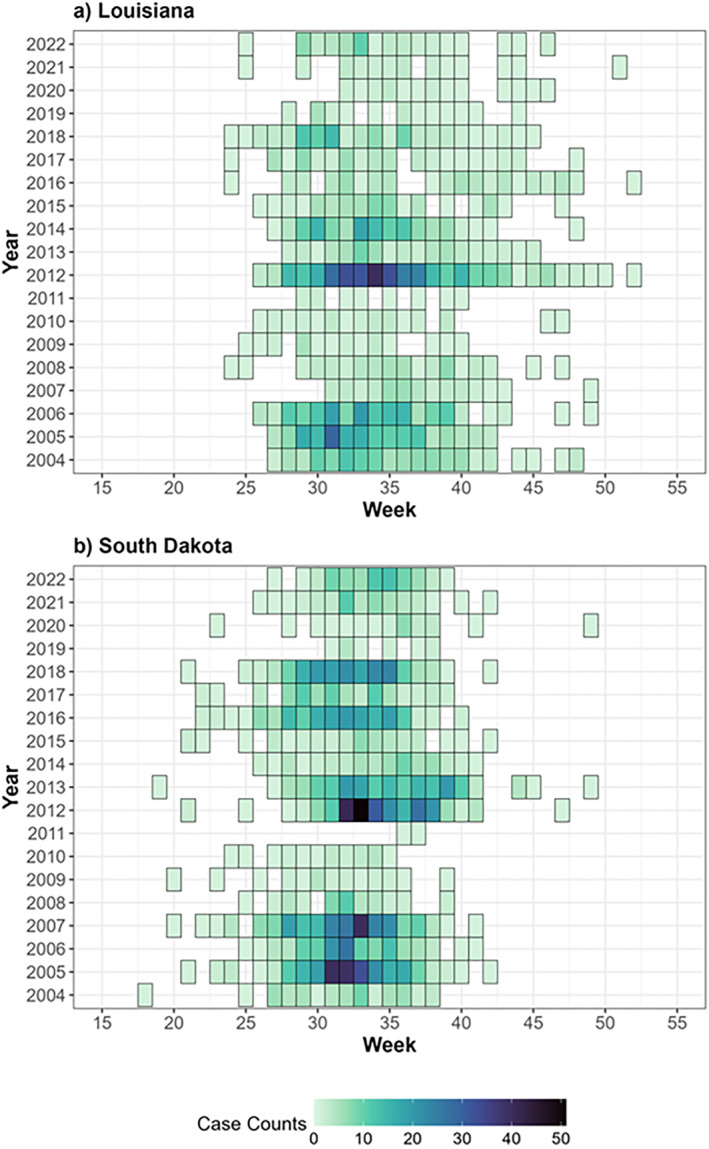
Occurrence of WNV in Louisiana (a) and South Dakota (b) from 2004 to 2022. The graphs depict the temporal distribution of WNV cases by week and year. In South Dakota, cases were compiled from WNV fever, neuroinvasive disease, and asymptomatic blood donations, whereas in Louisiana, only cases of fever and neuroinvasive disease were recorded.

The temporal dynamics of WNV transmission (Figure 3) reveal distinct seasonal patterns between Louisiana and South Dakota. In Louisiana, the transmission season extends from May through October, with peak activity occurring between July and September. This prolonged transmission period aligns with Louisiana's persistent warm, humid conditions, which sustain mosquito activity for longer. Conversely, South Dakota's WNV transmission season is more compressed, spanning June to September, with peak incidence concentrated in July and August. The shorter season in South Dakota reflects the constraints of its continental climate, where cooler spring and fall temperatures curtail mosquito activity earlier and later in the year. Notably, both regions exhibit significant year‐to‐year variability in the timing and intensity of WNV transmission, as evidenced by the fluctuating patterns across years. In Louisiana, variability is marked by occasional years of heightened transmission, most notably in 2005, 2006, and 2012, while other years, such as 2011, exhibit lower and more sporadic transmission. Similarly, South Dakota shows substantial variation, with high WNV activity in years like 2005, 2007, and 2012, contrasted by low to moderate transmission in others. This variability highlights the influences of annual climatic fluctuations and other environmental factors on mosquito populations and virus transmission cycles across both states.

### 2.2 Data Sources

Data for this study were obtained from two primary sources: human WNV case reports from state health departments and meteorological data from the Gridded Surface Meteorological (gridMET) database (Table 2). The WNV case data, shared under agreements with the Louisiana and South Dakota state health departments, included 1,465 and 1,822 records, respectively, covering 19 years from 2004 to 2022. These records encompassed neuroinvasive and non‐neuroinvasive cases, including confirmed WNV fever and neuroinvasive disease in both states. In addition, South Dakota's data set included incidental detections identified through blood donation screening, whereas Louisiana's data set did not include any such cases. For viremic blood donors, the reference date was the date of blood donation. Although the exact infection date is unknown, WNV nucleic acid is generally detectable for approximately 7–8 days postinfection (Busch et al., 2008; Zou et al., 2010). Consequently, the donation date should be within about 1 week of exposure and is unlikely to introduce a substantial bias in our temporal analyses. This approach is consistent with prior studies that use symptom onset for clinical cases and donation date for viremic donors, as both are considered reliable proxies of the time of infection (Rudolph et al., 2014).

**Table 2:** Climate Variables (Temperature, Vapor Pressure Deficit, Precipitation, Relative Humidity, and Wind Speed) From the Gridded Surface Meteorological (gridMET) Database

Data set	Variable	Description
Gridded meteorological data (GridMET)	tminc tmeanc tmaxc	Temperature (°C)
	pr	Daily total precipitation (mm)
	rmean	Mean Relative humidity (%)
	vpd	Mean Vapor pressure deficit (kPA)
	vs	Wind Speed at 10 m (m/s)
Human WNV Cases	Case counts	Neuroinvasive, non‐neuroinvasive, asymptomatic blood donations

*Note*. Human WNV case data covering the period from 2004 to 2022, including neuroinvasive and non‐neuroinvasive cases, as well as asymptomatic blood donations (the latter only in South Dakota), were provided by the Louisiana and South Dakota State Departments of Health.

According to CDC data from 2004 to 2022 (CDC, 2025), in Louisiana, 34% of reported WNV cases were classified as neuroinvasive, 58% as non‐neuroinvasive, and 8% were identified through blood donation. By contrast, in South Dakota, 20% of cases were neuroinvasive, 72% were non‐neuroinvasive, and 8% were identified through blood donation. A summary figure illustrating these proportions across the study years in each state is provided in Supporting Information S1.

We excluded data from the epidemic years of 2002 and 2003 to focus on the period after the initial outbreak of WNV throughout the United States. These early years exhibited unusually high incidence rates due to the introduction and rapid spread of the virus, which may not accurately represent the established transmission dynamics. By starting our analysis from 2004, we aim to examine WNV transmission under more stabilized, endemic conditions, allowing for a more explicit assessment of climate‐driven epidemiological patterns. All cases were initially reported at the individual level, referenced by the date of symptom onset for symptomatic cases or the date of donation for blood donors. Due to a lack of travel status information, each case was attributed to the individual's county of residence. For this study, we aggregated the data to weekly counts at the county level. The data were collected for routine public health surveillance, and only aggregated county‐level summaries were used, with no personal identifiers, exempting the study from institutional review board oversight.

Meteorological data were sourced from the gridMET data set (Abatzoglou, 2013), which integrates the NASA North American Land Data Assimilation System (NLDAS) with the Parameter‐elevation Regressions on Independent Slopes Model (PRISM) to provide high‐resolution, daily climatic variables across the contiguous United States at a 4‐km spatial scale. Seven climatic variables were extracted: mean, minimum, and maximum temperature (°C), vapor pressure deficit (kPa), total precipitation (mm), relative humidity (%), and wind speed (m/s). These data, processed via a Google Earth Engine script, were aggregated at the county level for 2000–2023 and formatted for statistical analysis (Nekorchuk et al., 2024). To facilitate spatial and temporal analysis, weekly WNV case data were dichotomized to indicate the presence or absence of at least one case per county. This binary classification allowed logistic regression models to assess the relationships between WNV occurrence and environmental variables, simplifying the analysis while maintaining the capacity to detect spatial and temporal patterns (Davis et al., 2018; Wimberly et al., 2022).

### 2.3 Analysis

We used a logistic regression framework to model weekly WNV occurrence at the county level, with a binary outcome indicating whether at least one WNV case was reported in a given county‐week (Wimberly et al., 2022). The model consisted of four primary components: (a) county‐level fixed effects, (b) a cyclical seasonal term, (c) anomalized daily meteorological variables with distributed lag effects, and (d) a binary indicator for WNV presence or absence. Formally, the model can be written as:

$$\text{logit}\left(p_{i,t}\right)=c_{0i}+\text{cyc}\left(w\right)+\sum_{k=1}^{K}\sum_{l=0}^{L}S_{k}\left(l\right)a_{i,t-l}$$

Where $p_{i,t}$​ is the probability of observing one or more WNV cases in county i during week t; $c_{0i}$​ denotes the fixed effect for county i, which accounts for time‐invariant local conditions such as population density, vector‐control funding, and healthcare access that shape baseline WNV risk (Davis et al., 2018; Wimberly et al., 2022); cyc(w) is a cyclical seasonal function of the epidemiological week *w*, capturing predictable annual fluctuations in mosquito populations and virus transmission (Nekorchuk et al., 2024; Wimberly et al., 2022); $a_{i,l-l}$ refers to the anomalized value of meteorological variable k(k=1,…,K) for county i, lagged l days before week t.

Daily anomalies were calculated by subtracting each day's observed meteorological value from its long‐term seasonal expectation (2000–2023), thereby highlighting atypical conditions such as heat waves and droughts (Wimberly et al., 2022). $S_{k}\left(l\right)$ *,* is a thin‐plate spline that flexibly models how the effect of meteorological variable k, varies across l (up to L days) and week of year w (Gasparrini et al., 2010).

The county‐level fixed effects $c_{0i}$​ isolate how within‐county fluctuations in climate anomalies influence WNV risk by controlling for stable spatial differences (e.g., rural vs. urban settings, healthcare infrastructure), thereby reducing potential confounding from unobserved factors (Davis et al., 2018; Wimberly et al., 2022). The cyclical term cyc(w) captures the well‐established seasonal pattern in mosquito abundance and virus activity, enabling the model to distinguish these regular seasonal effects from the short‐term influences of anomalous weather (Nekorchuk et al., 2024; Wimberly et al., 2022).

We defined meteorological anomalies as the raw residuals from a county‐specific, cyclical spline model that describes the expected climatological curve for each variable. Specifically, for every county, we fitted a generalized additive model to the full 2000–2023 record, using a cyclic thin‐plate spline of day‐of‐year to capture typical climatological conditions. An anomaly on a given day is therefore the difference between that day's observed value and the model‐derived long‐term daily climatological mean. By adopting the broader 2000–2023 baseline, we ensured that the reference climatology spans the entire human WNV case analysis window (2004–2022) plus additional years, yielding more stable estimates without excluding any study observations from the baseline. This framework retains sensitivity to both acute extremes (e.g., short‐lived heat waves) and protracted deviations (e.g., multi‐year droughts), allowing us to test how immediate and lagged weather conditions shape WNV transmission dynamics.

Distributed lag effects were utilized to capture how climatic conditions preceding the current week, sometimes by several months, can shape mosquito dynamics and viral amplification (Gasparrini et al., 2010; Teller et al., 2016). Rather than choosing a fixed lag length, we used smooth thin‐plate spline functions, $S_{k}\left(l\right)$ to let the data identify the most relevant time windows over which temperature, precipitation, humidity, and wind anomalies influence WNV occurrence (Davis et al., 2018; Nekorchuk et al., 2024; Smith et al., 2020).

We employed a multi‐model comparison approach, systematically varying combinations of climatic variables. Our analysis began with a base model that incorporated only a seasonal cyclical function, without meteorological covariates, followed by single‐variable models, each including only one temperature metric (mean, minimum, or maximum). Two‐variable models expanded upon these temperature metrics by adding either humidity (relative humidity or vapor pressure deficit [VPD]), precipitation, or wind speed. Three‐variable models further integrated one humidity variable alongside precipitation, and optionally, wind speed was added to generate additional three‐ and four‐variable configurations. To minimize collinearity and maintain interpretability, we structured the models to avoid the simultaneous inclusion of strongly correlated climatic variables. Specifically, no model included more than one temperature metric (mean, minimum, or maximum), nor were VPD and relative humidity ever combined in the same model. By limiting each model to a single variable per climatic dimension, temperature, humidity, precipitation, and optionally wind speed, we reduced redundancy and facilitated clear comparisons across diverse model configurations.

Our primary objective was to characterize the relative influence of individual climatic factors on WNV cases rather than to identify a singular optimal explanatory model. Therefore, mild to moderate collinearity was deemed acceptable and was not expected to compromise the validity of our findings (Dormann et al., 2013).

We evaluated model performance using Akaike's Information Criterion (AIC) and the Bayesian Information Criterion (BIC). AIC identifies the most parsimonious models by balancing model fit and parameter complexity, grounded in Kullback–Leibler information, emphasizing explanatory accuracy, and recognizing that no single model is strictly true (Burnham et al., 2011; Davis et al., 2018; Groen et al., 2017). In contrast, BIC assumes the existence of a single “true” model within the candidate set and applies a stricter penalty for additional parameters (Burnham et al., 2011). Given that ecological models are generally viewed as approximations of complex environmental processes rather than literal representations, AIC is particularly suitable for ecological research because it facilitates multi‐model inference and model averaging, aligning closely with practical goals of ecological understanding and forecasting (Burnham et al., 2011; Johnson & Omland, 2004). Although we primarily relied on AIC for model selection due to these considerations, we also included BIC comparisons to provide additional transparency and context.

The modeling approaches utilized in this study were derived from the Arbovirus Mapping and Prediction (ArboMAP) system, initially developed in 2016 for analyzing WNV transmission dynamics in South Dakota (Davis et al., 2018). ArboMAP integrates environmental data with public health surveillance to generate weekly, county‐level forecasts of WNV risk. The system has been successfully implemented across South Dakota and Louisiana public health agencies, demonstrating its utility in routine WNV surveillance and forecasting (Nekorchuk et al., 2024). Building on this existing framework, this study incorporated additional climatic variables, specifically wind data, to enhance our understanding of how various climatic factors collectively influenced the dynamics of WNV cases.

## 3 Results

The multi‐model comparison of climatic variables revealed substantial differences in model performance for explaining WNV cases in both Louisiana and South Dakota. In each state, wind speed consistently improved model fit, underscoring its value as an explanatory variable (Table 3). In Louisiana, the best‐performing model (tminc‐vpd‐pr‐vs; AIC = 6,570, AIC weight = 0.996) incorporated minimum temperature, vapor pressure deficit (VPD), precipitation, and wind speed. This model outperformed the next‐best model (tminc‐rmean‐pr‐vs; AIC = 6,581, AIC weight = 0.004), which substituted relative humidity for VPD, by a ΔAIC of 11. Notably, the best‐fitting model that excluded wind speed (tminc‐vpd‐pr; AIC = 6,766, AIC weight = 0.000) had a substantially higher AIC (ΔAIC = 196), reinforcing the critical role of wind speed in model performance. Similarly, in South Dakota, the top‐performing model (tmaxc‐vpd‐pr‐vs; AIC = 8,380, AIC weight = 0.982) included maximum temperature, VPD, precipitation, and wind speed. It outperformed the second‐best model (tmaxc‐rmean‐pr‐vs; AIC = 8,388, AIC weight = 0.018), which replaced VPD with relative humidity, by a ΔAIC of 8. The best model without wind speed (tmaxc‐vpd‐pr, AIC = 8,407, AIC weight = 0.000) yielded a higher AIC (ΔAIC = 27), again indicating a poorer fit. Overall, wind speed emerged as a key climatic variable in both states, consistently improving model performance by lowering AIC values and increasing AIC weights when included.

**Table 3:** Multi‐Model Comparison of Akaike Information Criterion (AIC) Values for Climate Variables Influencing WNV Cases in Louisiana and South Dakota

Louisiana				South Dakota			
Climate model	AIC	∆AIC	AICWt	Climate model	AIC	∆AIC	AICWt
tminc‐vpd‐pr‐vs	6,570	0	0.996	tmaxc‐vpd‐pr‐vs	8,380	0	0.982
tminc‐rmean‐pr‐vs	6,581	11	0.004	tmaxc‐rmean‐pr‐vs	8,388	8	0.018
tminc‐vpd‐vs	6,587	17	0	tmeanc‐vpd‐pr‐vs	8,399	19	0
tminc‐rmean‐vs	6,588	18	0	tmeanc‐rmean‐pr‐vs	8,404	24	0
tmeanc‐vpd‐pr‐vs	6,614	44	0	tmaxc‐vpd‐pr	8,407	27	0
tminc‐pr‐vs	6,623	53	0	tmaxc‐rmean‐pr	8,412	32	0
tmeanc‐vpd‐vs	6,626	56	0	tmeanc‐vpd‐pr	8,415	35	0
tmeanc‐rmean‐pr‐vs	6,675	105	0	tmeanc‐rmean‐pr	8,426	46	0
tmeanc‐rmean‐vs	6,684	114	0	tminc‐vpd‐pr‐vs	8,446	66	0
tmaxc‐rmean‐pr‐vs	6,706	136	0	tmaxc‐rmean‐vs	8,449	69	0
tmaxc‐vpd‐pr‐vs	6,716	146	0	tminc‐rmean‐pr‐vs	8,455	75	0
tmaxc‐rmean‐vs	6,732	162	0	tmeanc‐rmean‐vs	8,462	82	0
tmaxc‐vpd‐vs	6,732	162	0	tmeanc‐pr‐vs	8,467	87	0
tminc‐vs	6,757	187	0	tmaxc‐vpd‐vs	8,473	93	0
tmeanc‐vs	6,759	189	0	tmaxc‐pr‐vs	8,475	95	0
tminc‐vpd‐pr	6,766	196	0	tminc‐vpd‐pr	8,477	97	0
tmaxc‐vs	6,767	197	0	tminc‐rmean‐pr	8,479	99	0
tminc‐vpd	6,770	200	0	tmeanc‐vpd‐vs	8,487	107	0
tminc‐pr	6,772	202	0	tminc‐pr‐vs	8,487	107	0
tminc‐rmean	6,773	203	0	tmaxc‐vpd	8,507	127	0
tminc‐rmean‐pr	6,775	205	0	tmeanc‐pr	8,513	133	0
tmaxc‐pr‐vs	6,782	212	0	tmeanc‐vpd	8,522	142	0
tmeanc‐pr‐vs	6,799	229	0	tminc‐pr	8,523	143	0
tmeanc‐vpd‐pr	6,823	253	0	tminc‐rmean‐vs	8,526	146	0
tmeanc‐vpd	6,824	254	0	tmaxc‐pr	8,532	152	0
tmeanc‐rmean‐pr	6,861	291	0	tmaxc‐rmean	8,535	155	0
tmeanc‐rmean	6,863	293	0	tmaxc‐vs	8,536	156	0
tmeanc‐pr	6,887	317	0	tmeanc‐rmean	8,551	171	0
tmaxc‐rmean	6,903	333	0	tminc‐vpd‐vs	8,562	182	0
tmaxc‐rmean‐pr	6,906	336	0	tmeanc‐vs	8,562	182	0
tmaxc‐vpd	6,907	337	0	tminc‐vpd	8,565	185	0
tmaxc‐vpd‐pr	6,907	337	0	tminc‐vs	8,574	194	0
tmaxc‐pr	6,913	343	0	tmaxc	8,576	196	0
tminc	7,299	729	0	tminc‐rmean	8,601	221	0
tmeanc	7,431	861	0	tmeanc	8,606	226	0
tmaxc	7,439	869	0	tminc	8,616	236	0
base	7,614	1,044	0	base	8,746	366	0

*Note*. Lower AIC values indicate better model fit. ΔAIC shows the difference from the best model, and AIC weights represent the probability of each model being the best.

Additionally, BIC values were examined in parallel with our AIC analysis. Although overall patterns were broadly consistent, the top‐ranked models differed due to BIC's more substantial penalty for model complexity as sample size increases. In Louisiana, the best‐performing BIC model (tminc‐vpd‐vs; BIC = 7,202, BIC weight = 0.622), which included minimum temperature, vapor pressure deficit, and wind speed, yielded a substantially lower BIC than the best model without wind speed (tminc‐vpd; BIC = 7,370, BIC weight = 0.000; ΔBIC = 168). Unlike the AIC ranking, precipitation was not included in Louisiana's top‐ranked BIC model. In South Dakota, the lowest BIC value (9,118, BIC weight = 0.678) was achieved by tmaxc‐vpd‐pr, whereas the second‐best model (tmaxc‐vpd‐pr‐vs; BIC = 9,120, BIC weight = 0.249) had a nearly identical BIC (ΔBIC = 2). Under the conventional “rule of thumb” for BIC comparisons (ΔBIC ≤ 2), these two South Dakota models are considered competing models (Burnham & Anderson, 2002). The observed discrepancies between AIC and BIC rankings largely reflect the fundamental differences in their approaches to model selection (see Section 2). Given our focus on explanatory analysis, and recognizing that no single model can be considered strictly true, we emphasize AIC as the primary model criterion, with BIC results used secondarily to provide additional context. All BIC values for our models are provided in Supporting Information S1.

**Figure 4:**
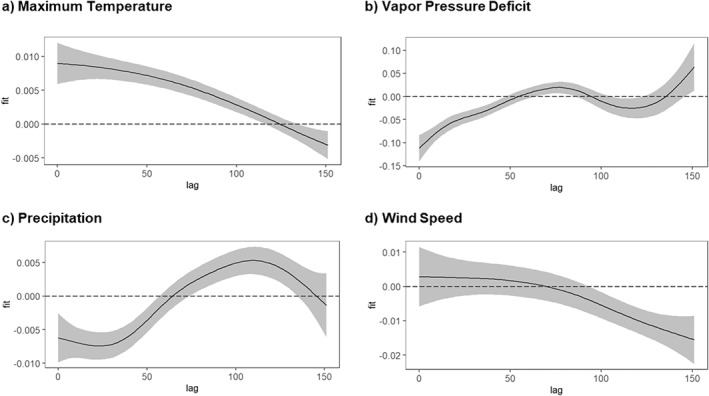
Distributed lag effects of climatic variables on WNV cases in Louisiana. *Y*‐axis values are on the log‐odds scale from our logistic model, and the shaded areas represent 95% confidence intervals. Panel labels indicate each climate variable under study. (a) Minimum temperature (tmin), (b) Vapor pressure deficit (vpd), (c) Precipitation (pr), and (d) Wind speed (vs).

In Louisiana, the optimal model's distributed lag effects (Figure 4) revealed that lower‐than‐normal minimum temperatures over the preceding 100 days were significantly associated with increased WNV cases. Increased case counts also correlated with drier atmospheric conditions, as indicated by higher VPD values observed 20–110 days prior. Precipitation exhibited a delayed positive association with WNV cases; rainfall occurring 20–120 days earlier was linked to increased cases. Higher‐than‐normal wind speeds over the preceding 30–150 days were associated with fewer WNV cases, suggesting a negative influence on virus transmission.

**Figure 5:**
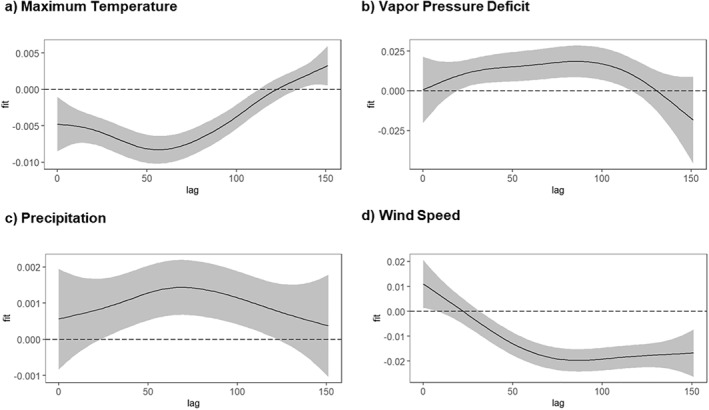
Distributed lag effects of climatic variables on WNV cases in South Dakota. *Y*‐axis values are on the log‐odds scale from our logistic model, and the shaded areas represent 95% confidence intervals. Panel labels indicate each climate variable under study. (a) Maximum temperature (tmin), (b) Vapor pressure deficit (vpd), (c) Precipitation (pr), and (d) Wind speed (vs).

In South Dakota, the optimal model's distributed lag effects (Figure 5) demonstrated that higher‐than‐normal maximum temperatures were associated with increased WNV cases, with the effect gradually diminishing over time yet remaining positive. VPD exhibited a negative relationship with WNV cases in the first 2 months. Precipitation initially had a negative effect on WNV cases from 0 to 60 days, followed by a delayed positive effect approximately 100 days after rainfall. Wind speed consistently displayed a delayed negative impact, with the most pronounced reductions in WNV cases observed between 60 days and the end of the lag period, implying that higher wind speeds are inhibitory. Overall, the findings in both states demonstrate that each climatic variable exerts a distinct, time‐dependent influence on WNV transmission across specific lag periods.

## 4 Discussion

Climatic factors, including temperature, precipitation, and humidity, exhibited different influences on WNV incidence in Louisiana and South Dakota, underscoring the complexity of climatic effects and the importance of regional context (Morin & Comrie, 2013; Wimberly et al., 2014). The observed differences in temperature effects reflect the nonlinear relationship between temperature and WNV transmission, which peaks between about 23°C and 26°C (Shocket et al., 2020). In Louisiana's subtropical climate, cooler‐than‐normal temperatures bring daily temperature distributions closer to the optimal range, particularly during summer, thereby enhancing mosquito development rates and viral replication and increasing WNV cases. Conversely, South Dakota's continental climate has daily temperature distributions near or slightly below the optimal range, particularly during the amplification period in late spring and early summer. As a result, warmer‐than‐usual temperatures can shift conditions closer to the optimum, thereby increasing WNV transmission rates. Although the best models are driven by minimum temperature in Louisiana and maximum temperature in South Dakota, temperature effects on WNV reflect the full diurnal range of temperatures, rather than just the daily extremes. Our results emphasize that understanding these deviations is crucial for explaining spatially heterogeneous effects of temperature on WNV transmission dynamics (Mordecai et al., 2017; Shocket et al., 2020). The influence of humidity on mosquito biology and WNV transmission remains less clearly defined, but an optimal humidity range likely exists. Our findings support this idea, as vapor pressure deficit (an inverse measure of humidity) showed opposite relationships with WNV cases across regions: it was negatively correlated with WNV incidence in humid Louisiana but positively correlated in drier South Dakota. This regional difference underscores that the effects of humidity anomalies on WNV transmission vary according to baseline climatic environments.

In South Dakota, high precipitation was associated with a reduction in WNV cases for up to 2 months, a pattern not observed in Louisiana. This suggests that flushing effects inhibiting larval development may be more significant in South Dakota (Gardner et al., 2012; Koenraadt & Harrington, 2008). While a short‐term precipitation deficit may contribute to drier conditions, true drought involves broader hydrometeorological processes and can be classified in several ways: meteorological, hydrological, or agricultural (Svoboda & Fuchs, 2016). Lower precipitation levels can concentrate mosquitoes and avian hosts around limited water sources or promote the presence of smaller, more suitable larval habitats (Landesman et al., 2007; Shaman et al., 2005). Consequently, periods of reduced precipitation can create conditions favorable for *Cx*. *tarsalis*, which thrives in drier environments and has been associated with increased WNV activity during drought‐like conditions (Paull et al., 2017). This mechanism aligns with our finding of a negative relationship between precipitation and WNV cases at shorter lag periods in South Dakota. Over longer lags, however, precipitation was positively associated with WNV occurrence in both Louisiana and South Dakota.

While previous studies on WNV incidence have primarily focused on temperature, precipitation, and humidity, wind speed has often been overlooked (Groen et al., 2017; Shand et al., 2016; Smith et al., 2020; Stilianakis et al., 2016). Our study demonstrates that incorporating wind speed into time series models consistently improved model performance in Louisiana and South Dakota, highlighting its role in shaping the environmental conditions associated with WNV cases. We found that higher‐than‐normal wind speeds were consistently associated with decreased reported WNV cases in both states. This inverse relationship may stem from the disruptive effects of higher wind speeds on mosquito flight and feeding behaviors; wind impedes mosquito dispersal and host‐seeking activity by limiting mosquitoes' ability to locate hosts (Adeleke et al., 2022; Gillies & Wilkes, 1981; Service, 1980). Consequently, reduced mosquito activity decreases human‐mosquito contact and subsequent virus exposure, ultimately influencing the spatial and temporal patterns of WNV incidence (Ma et al., 2022).

Our results demonstrate the broad applicability of wind speed as a significant factor in modeling WNV cases across regions with distinct environmental conditions. Despite differences in climate, ecosystems, vector and host species, and human geography between Louisiana and South Dakota, higher wind speeds consistently had a negative on WNV cases in both states. This suggests that wind may have more generalizable effects on mosquitoes, avian hosts, and virus transmission than other meteorological variables. Notably, the time lag before this effect was observed differed between the states: 90 days in South Dakota and 30 days in Louisiana, indicating that early‐season wind effects during the virus amplification phase in late spring and early summer may play a more crucial role in South Dakota. The consistent improvement in model performance with the inclusion of wind speed in both regions emphasizes the importance of considering wind speed when developing climate‐based models of WNV diverse geographic areas.

By focusing on two climatically distinct states, our study allowed for a detailed comparison of regional dynamics. Previous research examining weather effects on WNV, such as studies by Wimberly et al. (2014), Hahn et al. (2015), Gorris et al. (2023), and Moser et al. (2024) also investigated regional differences but primarily used temperature and precipitation to characterize historical weather patterns. Paull et al. (2017) included temperature and precipitation in their analysis of annual variation in WNV cases across all U.S. states. They considered extreme weather events, such as droughts and freezes, as well as variables related to population immunity. Our findings highlight that humidity and wind speed are important meteorological drivers of WNV transmission in addition to temperature and precipitation, and should be incorporated into future assessments.

In contrast to previous regional and national studies that conducted analyses at the county level using annual totals of human WNV cases and annual summaries of temperature and precipitation by month or season, our approach utilized weekly summaries of WNV data and distributed lags of daily meteorological data. This methodology allowed us to be sensitive to specific weather events, such as heatwaves and droughts, which can influence mosquito populations and WNV amplification. These acute events are often overlooked in national analyses that rely on annual summaries, where aggregation tends to smooth out the variability that drives outbreaks (Hahn et al., 2015; Wimberly et al., 2014). By employing weekly WNV data and daily weather variables, we could detect lagged relationships without constraining them to predefined periods, providing a more flexible and precise understanding of transmission dynamics. This aligns with the findings of Uelmen et al. (2021), who demonstrated that understanding spatial determinants of WNV dynamics requires fine‐scale data to capture the heterogeneity within local ecosystems, which is lost when data are aggregated at county and coarser resolutions.

While this study provides valuable insights into how regional climate influences WNV transmission, several limitations must be acknowledged. We used county‐level aggregated data, which may mask finer‐scale variations in mosquito populations and human cases (Hess et al., 2018; Uelmen et al., 2021). Reliance on reported WNV cases introduces potential bias from underreporting, asymptomatic infections, or misdiagnosis. However, using a dichotomized outcome (presence vs. absence of reported cases) helps mitigate this bias, as underreporting is more likely to be nondifferential with respect to climatic anomalies. Additionally, the county‐level resolution of the analysis may not capture localized microclimatic conditions that are critical for mosquito habitats and virus transmission (Boser et al., 2021; Erraguntla et al., 2021; Wimberly et al., 2020). Because we analyzed relative climatic variations rather than absolute microclimates, this limitation likely exerts only a modest effect on our core findings. Nonetheless, incorporating station‐based observations or downscaled climate models (Faridah et al., 2022; Sauer et al., 2022) could further enhance the characterization of environmental factors relevant to WNV spread. Another important limitation is the lack of entomological and ornithological data. This hinders our ability to determine whether climate primarily affects WNV through direct conditions (e.g., temperature or humidity) or indirectly via shifts in vector or host populations (Davis et al., 2017). As Table 1 shows, the most abundant bird species differ significantly between Louisiana and South Dakota, underscoring how local avian communities may shape WNV dynamics. Yet, obtaining consistent avian and mosquito data with sufficient spatial and temporal resolution is challenging, so our models emphasized climatic variables. More localized analyses could incorporate high‐resolution mosquito abundance, infection data, and longitudinal bird surveillance or banding information to build more comprehensive mechanistic models of mosquito behavior, habitat characteristics, and avian ecology (Bhowmick et al., 2024; Ewing et al., 2019; Ferraguti et al., 2021; Kain & Bolker, 2019). Such detailed data sets would help clarify the distinction between direct and indirect climatic influences, particularly in regions that differ markedly in climate and ecological context, such as Louisiana and South Dakota. Finally, while wind speed improved model fit in our retrospective analyses, further research is needed to assess whether it can enhance real‐time WNV forecasting. The biological plausibility of wind's inhibitory effect on mosquito flight and disease transmission is apparent; however, validating wind‐based predictions against new observations remains essential. Such efforts would help clarify whether wind speed can be incorporated into operational surveillance and modeling systems for routine disease forecasts.

As global temperatures rise and regional weather patterns shift, the geographic ranges of mosquito vector and the timing of transmission seasons are expected to change accordingly. While warming could increase WNV risk in northern states like South Dakota, higher temperatures in southern regions such as Louisiana may ultimately reduce transmission if they exceed the optimal range. Changes in precipitation, humidity, and wind speed, which are geographically variable and difficult to forecast, further complicate assessments of future disease trends. Climate‐driven shifts in avian migration timing and routes, along with human responses such as altered outdoor activities and water‐management practices, may also reshape local WNV dynamics (Paz, 2015; Schwartz et al., 2025). Recognizing these multifactorial effects highlights the uncertainties inherent in anticipating how climate change may reshape WNV transmission. Addressing these uncertainties will require integrative research that combines multiple environmental risk factors across diverse ecological settings, thereby refining predictions of climate‐driven impacts on WNV and other vector‐borne diseases.

## Conflict of Interest

The authors declare no conflicts of interest relevant to this study.

## Supporting information

Supporting Information S1

## Data Availability

Public health surveillance data on human West Nile virus cases are collected and maintained by the Louisiana Department of Health and the South Dakota Department of Health. They are not publicly distributable due to confidentiality constraints. Researchers interested in accessing these data may contact each state's Department of Health directly to request data through a formal data‐sharing agreement. Details on the data request process, the required agreements, and any additional constraints can be obtained by reaching out to: Louisiana Department of Health: https://ldh.la.gov/bureau‐of‐infectious‐diseases/west‐nile‐virus
South Dakota Department of Health: https://doh.sd.gov/topics/diseases/infectious/reportable‐communicable‐diseases/west‐nile‐virus/ The ArboMAP system is under active development. The latest version of the code, along with a user guide and simulated test data, is available through the EcoGRAPH GitHub (https://github.com/EcoGRAPH) repository https://github.com/EcoGRAPH/ArboMAP_multivariable and on Zenodo (Bump et al., 2025; https://doi.org/10.5281/zenodo.15183297). Meteorological data from GridMET are publicly available at https://www.climatologylab.org/gridmet.html, and county‐level summaries of these data can be accessed or downloaded via a Google Earth Engine app (https://dawneko.users.earthengine.app/view/arbomap‐gridmet).
