# Supplementary material for: Integrating Wind Speed Into Climate‐Based West Nile Virus Models: A Comparative Analysis in Two Distinct Regions
Source: Geohealth. 2025 Jul 5;9(7):e2024GH001320. doi: 10.1029/2024GH001320 (PMC12228046; doi:10.1029/2024GH001320)
Supplement: Supplementary file 1 — Supporting Information S1 [file GH2-9-e2024GH001320-s001.docx]

*AGU GeoHealth*

Supporting Information for

**Integrating Wind Speed into Climate-Based West Nile Virus Models: A Comparative Analysis in Two Distinct Regions**

**Eric R. Bump^1^, Anita Bharadwaja^2^, Sean Simonson^3^, Emma Ortega^3^, Michael C. Wimberly^1^**

**^1^University of Oklahoma, ^2^South Dakota Department of Health, ^3^Louisiana Department of Health**

**Contents of this file**

Table S1

Figure S1

**Introduction**

These Supplemental Materials present additional analyses and data that expand on and support our primary findings. Table S1 compares multiple climate models using Akaike Information Criterion (AIC) and Bayesian Information Criterion (BIC) to explore how different climate variables affect West Nile virus (WNV) cases in Louisiana and South Dakota. In addition to the AIC and BIC values, the table reports ΔAIC, ΔBIC, and the associated weights, providing insight into each model’s relative likelihood of being the best fit. Figure S1 shows the annual proportions of WNV cases by disease classification and blood donor status, revealing how the relative distribution of neuroinvasive, febrile, and donor-identified cases has shifted over time in both states.

| Louisiana | | | | | | | South Dakota | | | | | | | |
| --- | --- | --- | --- | --- | --- | --- | --- | --- | --- | --- | --- | --- | --- | --- |
| Climate Variable Model | **AIC** | **∆AIC** | **AICWt** | **BIC** | **∆BIC** | **BICWt** | | **Climate Variable Model** | **AIC** | **∆AIC** | **AICWt** | **BIC** | **∆BIC** | **BICWt** |
| tminc-vpd-pr-vs | 6570 | 0 | 0.996 | 7236 | 34 | 0.000 | | **tmaxc-vpd-pr-vs** | 8380 | 0 | 0.982 | 9120 | 2 | 0.249 |
| tminc-rmean-pr-vs | 6581 | 11 | 0.004 | 7247 | 45 | 0.000 | | **tmaxc-rmean-pr-vs** | 8388 | 8 | 0.018 | 9128 | 10 | 0.005 |
| tminc-vpd-vs | 6587 | 17 | 0.000 | 7202 | 0 | 0.622 | | **tmeanc-vpd-pr-vs** | 8399 | 19 | 0.000 | 9139 | 21 | 0.000 |
| tminc-rmean-vs | 6588 | 18 | 0.000 | 7203 | 1 | 0.378 | | **tmeanc-rmean-pr-vs** | 8404 | 24 | 0.000 | 9151 | 33 | 0.000 |
| tmeanc-vpd-pr-vs | 6614 | 44 | 0.000 | 7280 | 78 | 0.000 | | **tmaxc-vpd-pr** | 8407 | 27 | 0.000 | 9118 | 0 | 0.678 |
| tminc-pr-vs | 6623 | 53 | 0.000 | 7238 | 36 | 0.000 | | **tmaxc-rmean-pr** | 8412 | 32 | 0.000 | 9123 | 5 | 0.056 |
| tmeanc-vpd-vs | 6626 | 56 | 0.000 | 7241 | 39 | 0.000 | | **tmeanc-vpd-pr** | 8415 | 35 | 0.000 | 9126 | 8 | 0.012 |
| tmeanc-rmean-pr-vs | 6675 | 105 | 0.000 | 7341 | 139 | 0.000 | | **tmeanc-rmean-pr** | 8426 | 46 | 0.000 | 9137 | 19 | 0.000 |
| tmeanc-rmean-vs | 6684 | 114 | 0.000 | 7300 | 98 | 0.000 | | **tminc-vpd-pr-vs** | 8446 | 66 | 0.000 | 9187 | 69 | 0.000 |
| tmaxc-rmean-pr-vs | 6706 | 136 | 0.000 | 7373 | 171 | 0.000 | | **tmaxc-rmean-vs** | 8449 | 69 | 0.000 | 9160 | 42 | 0.000 |
| tmaxc-vpd-pr-vs | 6716 | 146 | 0.000 | 7383 | 181 | 0.000 | | **tminc-rmean-pr-vs** | 8455 | 75 | 0.000 | 9196 | 78 | 0.000 |
| tmaxc-rmean-vs | 6732 | 162 | 0.000 | 7347 | 145 | 0.000 | | **tmeanc-rmean-vs** | 8462 | 82 | 0.000 | 9173 | 55 | 0.000 |
| tmaxc-vpd-vs | 6732 | 162 | 0.000 | 7347 | 145 | 0.000 | | **tmeanc-pr-vs** | 8467 | 87 | 0.000 | 9178 | 60 | 0.000 |
| tminc-vs | 6757 | 187 | 0.000 | 7357 | 155 | 0.000 | | **tmaxc-vpd-vs** | 8473 | 93 | 0.000 | 9184 | 66 | 0.000 |
| tmeanc-vs | 6759 | 189 | 0.000 | 7359 | 157 | 0.000 | | **tmaxc-pr-vs** | 8475 | 95 | 0.000 | 9186 | 68 | 0.000 |
| tminc-vpd-pr | 6766 | 196 | 0.000 | 7381 | 179 | 0.000 | | **tminc-vpd-pr** | 8477 | 97 | 0.000 | 9188 | 70 | 0.000 |
| tmaxc-vs | 6767 | 197 | 0.000 | 7367 | 165 | 0.000 | | **tminc-rmean-pr** | 8479 | 99 | 0.000 | 9190 | 72 | 0.000 |
| tminc-vpd | 6770 | 200 | 0.000 | 7370 | 168 | 0.000 | | **tmeanc-vpd-vs** | 8487 | 107 | 0.000 | 9198 | 80 | 0.000 |
| tminc-pr | 6772 | 202 | 0.000 | 7372 | 170 | 0.000 | | **tminc-pr-vs** | 8487 | 107 | 0.000 | 9198 | 80 | 0.000 |
| tminc-rmean | 6773 | 203 | 0.000 | 7373 | 171 | 0.000 | | **tmaxc-vpd** | 8507 | 127 | 0.000 | 9184 | 66 | 0.000 |
| tminc-rmean-pr | 6775 | 205 | 0.000 | 7390 | 188 | 0.000 | | **tmeanc-pr** | 8513 | 133 | 0.000 | 9190 | 72 | 0.000 |
| tmaxc-pr-vs | 6782 | 212 | 0.000 | 7397 | 195 | 0.000 | | **tmeanc-vpd** | 8522 | 142 | 0.000 | 9199 | 81 | 0.000 |
| tmeanc-pr-vs | 6799 | 229 | 0.000 | 7414 | 212 | 0.000 | | **tminc-pr** | 8523 | 143 | 0.000 | 9200 | 82 | 0.000 |
| tmeanc-vpd-pr | 6823 | 253 | 0.000 | 7438 | 236 | 0.000 | | **tminc-rmean-vs** | 8526 | 146 | 0.000 | 9237 | 119 | 0.000 |
| tmeanc-vpd | 6824 | 254 | 0.000 | 7424 | 222 | 0.000 | | **tmaxc-pr** | 8532 | 152 | 0.000 | 9209 | 91 | 0.000 |
| tmeanc-rmean-pr | 6861 | 291 | 0.000 | 7476 | 274 | 0.000 | | **tmaxc-rmean** | 8535 | 155 | 0.000 | 9212 | 94 | 0.000 |
| tmeanc-rmean | 6863 | 293 | 0.000 | 7463 | 261 | 0.000 | | **tmaxc-vs** | 8536 | 156 | 0.000 | 9213 | 95 | 0.000 |
| tmeanc-pr | 6887 | 317 | 0.000 | 7487 | 285 | 0.000 | | **tmeanc-rmean** | 8551 | 171 | 0.000 | 9228 | 110 | 0.000 |
| tmaxc-rmean | 6903 | 333 | 0.000 | 7503 | 301 | 0.000 | | **tminc-vpd-vs** | 8562 | 182 | 0.000 | 9273 | 155 | 0.000 |
| tmaxc-rmean-pr | 6906 | 336 | 0.000 | 7521 | 319 | 0.000 | | **tmeanc-vs** | 8562 | 182 | 0.000 | 9239 | 121 | 0.000 |
| tmaxc-vpd | 6907 | 337 | 0.000 | 7507 | 305 | 0.000 | | **tminc-vpd** | 8565 | 185 | 0.000 | 9242 | 124 | 0.000 |
| tmaxc-vpd-pr | 6907 | 337 | 0.000 | 7522 | 320 | 0.000 | | **tminc-vs** | 8574 | 194 | 0.000 | 9251 | 133 | 0.000 |
| tmaxc-pr | 6913 | 343 | 0.000 | 7513 | 311 | 0.000 | | **tmaxc** | 8576 | 196 | 0.000 | 9209 | 91 | 0.000 |
| tminc | 7299 | 729 | 0.000 | 7883 | 681 | 0.000 | | **tminc-rmean** | 8601 | 221 | 0.000 | 9278 | 160 | 0.000 |
| tmeanc | 7431 | 861 | 0.000 | 8015 | 813 | 0.000 | | **tmeanc** | 8606 | 226 | 0.000 | 9240 | 122 | 0.000 |
| tmaxc | 7439 | 869 | 0.000 | 8023 | 821 | 0.000 | | **tminc** | 8616 | 236 | 0.000 | 9248 | 130 | 0.000 |
| base | 7614 | 1044 | 0.000 | 8158 | 956 | 0.000 | | **base** | 8746 | 366 | 0.000 | 9332 | 214 | 0.000 |

**Table S1.** A multi-model comparison of climate variables influencing West Nile virus (WNV) cases in Louisiana and South Dakota was evaluated using the Akaike Information Criterion (AIC) and Bayesian Information Criterion (BIC). The table also includes ΔAIC, ΔBIC, and weights based on AIC and BIC, illustrating each model’s relative likelihood of providing the best fit.

**Figure S1.** Annual proportions of WNV cases in Louisiana and South Dakota (2004–2022), categorized by disease classification, neuroinvasive, non‐neuroinvasive, and blood donor status. These data, drawn from the CDC (2025), highlight how the relative distribution of each classification category shifts over time in both states.
